# Percepções dos Participantes de Reabilitação Cardíaca sobre seus Comportamentos em Saúde e Necessidades de Informação durante a Pandemia COVID-19 no Brasil

**DOI:** 10.36660/abc.20210447

**Published:** 2022-03-21

**Authors:** Gabriela L.M Ghisi, Rafaella Z. Santos, Andrea S. Korbes, Cícero Augusto de Souza, Marlus Karsten, Paul Oh, Magnus Benetti

**Affiliations:** 1 Cardiovascular Prevention and Rehabilitation Program Toronto Rehabilitation Institute University Health Network Toronto Canadá Cardiovascular Prevention and Rehabilitation Program, Toronto Rehabilitation Institute, University Health Network, Toronto – Canadá; 2 Núcleo de Cardioncologia e Medicina do Exercício Centro de Ciências da Saúde e do Esporte Universidade do Estado de Santa Catarina Florianópolis SC Brasil Núcleo de Cardioncologia e Medicina do Exercício, Centro de Ciências da Saúde e do Esporte, Universidade do Estado de Santa Catarina, Florianópolis, SC – Brasil; 3 Instituto de Cardiologia de Santa Catarina São José SC Brasil Instituto de Cardiologia de Santa Catarina, São José, SC – Brasil

**Keywords:** Reabilitação Cardíaca, COVID-19, Letramento em Saúde, Determinação de Necessidades de Cuidados de Saúde, Inquéritos e Questionários

## Abstract

**Fundamento:**

A COVID-19 afetou como as pessoas recebem atendimento de saúde para várias doenças, inclusive doenças cardiovasculares.

**Objetivos:**

Examinar as percepções dos participantes de reabilitação cardíaca (RC) sobre seus comportamentos em saúde e necessidades de informação durante a pandemia da COVID-19 no Brasil.

**Métodos:**

Neste estudo transversal, um questionário de 27 itens elaborado pelos investigadores foi administrado online a participantes de dois programas de RC. As perguntas incluíam letramento em saúde (LS; usando a *Brief Health Literacy Screening Tool* - Breve ferramenta de triagem de letramento em saúde), uso de tecnologia, percepções antes e durante a pandemia da COVID-19, e necessidades de informações. Foram usados coeficiente de correlação de Pearson, testes t pareados e ANOVA, conforme apropriado. Um p <0,05 foi considerado estatisticamente significativo para todos os testes.

**Resultados:**

No total, 159 (25,5%) participantes de RC responderam ao questionário. Desses, 89,9% tinham LS limitado ou marginal, e 96,2% relataram ter acesso à internet de casa. Os pacientes se preocupam principalmente com a saúde de sua família e própria, além de como o coronavírus é perigoso para sua saúde e como mudou seu estilo de vida. Os participantes perceberam que a qualidade de seus comportamentos em saúde diminuiu significativamente durante a pandemia. A pandemia também mudou as necessidades de informações dos participantes de RC, já que novas necessidades surgiram, tais como, controle de níveis de ansiedade, manter a motivação para levar uma vida saudável durante a pandemia, e como a COVID-19 pode afetar sua condição de saúde. Participantes com LS adequado perceberam significativamente a gravidade da doença e tinham significativamente mais acesso a informações do que os pacientes com LS limitado.

**Conclusões:**

Nossos resultados destacaram o impacto da pandemia nas percepções dos participantes de RC em relação a seus comportamentos em saúde e necessidades de informação, que podem ser influenciados pelos níveis de LS.

## Introdução

O SARS-CoV-2 é um novo coronavírus identificado como a causa da doença por coronavírus 2019 (COVID-19) que começou em Wuhan, na China no final de 2019 e se espalhou pelo mundo.^1^ Mais de um ano após ser classificada como pandemia, o número de casos de COVID-19 confirmados em todo o mundo chegou a 147.000.000, sendo que o Brasil ficou em terceiro lugar entre os países com o número mais alto de casos confirmados e em segundo lugar no número de mortes.^2^ Por se tratar de um *patógeno* altamente contagioso, as pessoas no mundo todo estão tentando evitar infecção pela prática do distanciamento social,^3^ o que afetou a forma como trabalham, conectam-se com outros, e recebem assistência de saúde para várias doenças, incluindo doenças cardiovasculares (DCVs).^4^

Doenças cardiovasculares estão entre os principais fardos de doença e são a principal causa de mortalidade em todo o mundo, com mais de 80% dessas mortes ocorrendo em países de renda baixa e média,^5^ incluindo o Brasil.^6^ A reabilitação cardíaca (RC) é um modelo estabelecido de prevenção secundária que não só demonstrou ter eficiência clínica e financeira, como também pode reduzir significativamente os índices de hospitalização e mortalidade.^7 - 9^ Em geral, a RC é realizada em ambientes clínicos, com os pacientes visitando hospitais ou centros de reabilitação para participar de sessões de exercício e educação.^10 , 11^ Portanto, as medidas necessárias para controlar a transmissão generalizada da COVID-19 afetaram a realização da RC, sendo estimado que aproximadamente 4400 programas se fecharam em todo o mundo devido à COVID-19 e os serviços presenciais, cancelados.^12^

No Brasil, a COVID-19 afetou um sistema que já ficava abaixo do ideal,^11^ e os programas desenvolveram formas remotas e inovadoras para oferecer os componentes principais nesse momento tão delicado,^12 , 13^ seguindo diretrizes e recomendações locais.^14^ A velocidade alta em que essas mudanças ocorreram, as ameaças econômicas que os prestadores de serviços de saúde e seus programas sofreram, e a incapacidade de muitos pacientes de navegar no mundo virtual afetaram participantes de RC de maneira ainda não exploradas. Embora existam muitas publicações sobre o impacto da COVID-19 nessa população,^15 , 16^ até onde sabemos não há estudos sobre como os participantes de RC percebem seus comportamentos em saúde e sobre as informações de que eles precisam para continuar a adotar comportamentos que farão com que eles tenham uma saúde melhor. Isso é especialmente importante, pois o distanciamento social, a quarentena e ordens de confinamento afetam nosso estilo de vida e, em pacientes cardíacos que já são sedentários e têm fatores de risco devido a comportamentos ruins,^17 , 18^ essas medidas podem aumentar o risco de eventos agudos. Além disso, os efeitos indiretos da pandemia da COVID-19 na saúde mental em geral são uma preocupação constante,^19 - 21^ principalmente em indivíduos com doenças cardiovasculares, já que eles têm mais probabilidade de desenvolver problemas de saúde mental (tais como depressão),^22^ que estão associados a um risco duas vezes maior de mortalidade cardiovascular.^23^

Portanto, há uma necessidade urgente de monitorar pacientes cardíacos virtualmente e personalizar o cuidado preventivo, ajudando a esses indivíduos em sua recuperação e na prevenção de eventos recorrentes.^24 - 26^ Para desenvolver um programa de RC ideal durante a pandemia da COVID-19 e após ela, é importante entender as percepções e necessidades dos pacientes. Portanto, o objetivo deste estudo foi examinar as percepções dos participantes de RC em relação a seus comportamentos em saúde e necessidades de informação durante a pandemia da COVID-19 no Brasil.

## Métodos

### Desenho

Este é um estudo de desenho transversal. A aprovação ética foi obtida do Comitê de Ética em Pesquisa na Universidade do Estado de Santa Catarina (UDESC; Florianópolis, Brasil: 4.341.132). Os dados foram coletados entre dezembro de 2020 e abril de 2021.

### Configurações e Participantes

Uma amostra de conveniência de participantes de RC foi recrutada de dois programas públicos na Grande Florianópolis (Instituto de Cardiologia de Santa Catarina e Núcleo de Cardio-oncologia e Medicina do Exercício). Antes da pandemia, os pacientes frequentavam esses centros 3 vezes por semana para sessões de exercício de 1 hora supervisionadas por uma equipe multidisciplinar. Alguns desses participantes também estavam participando de sessões de educação, como parte de um projeto de pesquisa. Devido à COVID-19, ambos os programas foram fechados a partir de março de 2020 e as atividades não foram retomadas durante esta pesquisa. Os critérios de exclusão foram os seguintes: ser analfabeto ou portador de qualquer problema visual ou cognitivo que impediria o participante de preencher a pesquisa.

### Procedimentos

Havia 623 participantes de RC quando ambos os programas foram fechados devido à COVID-19. Todos eles foram contactados por telefone e convidados a participar desta pesquisa. Para os interessados, foi agendada uma segunda ligação para que dessem o consentimento informado por vídeo, que foi gravado conforme indicado pelo Comitê de ética em pesquisa. Os participantes preencheram a pesquisa online usando Google Docs durante uma chamada de vídeo com um integrante da equipe de pesquisa.

### Medidas

Um questionário de 27 perguntas foi elaborado pelos investigadores para examinar os objetivos deste estudo (Apêndice 1). O questionário foi dividido nas 5 seguintes seções: (1) características sociodemográficas, (2) letramento em saúde e uso de tecnologia, (3) percepções sobre a pandemia da COVID-19, (4) percepções sobre comportamentos em saúde e sentimentos antes e durante a pandemia da COVID-19, e (5) necessidades de informação durante a pandemia da COVID-19.

Os itens tinham opções de resposta única, múltipla escolha e respostas abertas. As percepções sobre a pandemia foram relatadas usando uma escala Likert que variava entre 1 = discordo totalmente e 5 = concordo totalmente. As percepções sobre os comportamentos em saúde e sentimentos durante a pandemia da COVID-19 foram relatadas usando uma escala Likert que variava entre 1 = ruim e 5 = excelente. Necessidades de informação específicas a tópicos de educação que poderiam ajudar pacientes a aderir a comportamentos saudáveis foram relatadas em uma escala Likert que variava entre 1 = não muito importante e 5 = muito importante. Uma pontuação média foi calculada e analisada por níveis de letramento, sendo que as pontuações mais altas indicavam necessidades de informação mais altas. Foram solicitados inputs de especialistas em RC antes de a pesquisa ser administrada.

Dados clínicos (indicação de RC e fatores de risco cardíacos) foram extraídos de prontuários médicos e características sociodemográficas (nível de escolaridade, renda familiar, mudança na renda familiar devido à COVID-19, estado civil, e número de pessoas que vivem na mesma residência) foram autorrelatados pelos participantes. O letramento em saúde foi avaliado usando-se a *Brief Health Literacy Screening Tool* (Breve ferramenta de triagem de letramento em saúde),^27^ que foi traduzida para o português pela equipe de pesquisa. Cada um dos 4 itens valia de 1 a 5 pontos, dependendo das respostas dos participantes, que poderiam variar entre 4 e 20. Pontuações de 4 a 12 pontos foram classificadas como letramento em saúde limitado, de 13 a 16, como letramento em saúde marginal, e de 17 a 20, como letramento em saúde adequado.

### Análise de dados

A análise estatística foi realizada utilizando-se o software SPSS Versão 27.0 (IBM Inc 2020, NYC). Foram usadas estatísticas descritivas para descrever as características socioeconômicas e clínicas dos participantes. As variáveis contínuas foram apresentadas como média e desvio padrão, e as variáveis categóricas foram apresentadas como números absolutos e porcentagens. A análise de qui-quadrado para variáveis categóricas e os testes t para variáveis contínuas foram usados para comparar proporções de respondentes nas várias características. Todas as respostas abertas foram codificadas. Os coeficientes de correlação de Pearson foram usados para determinar a associação entre letramento em saúde e nível de escolaridade, uso de tecnologia e características socioeconômicas, e letramento em saúde e percepções sobre a pandemia da COVID-19.

A normalidade da distribuição de dados foi testada usando-se o teste de Kolmogorov Smirnov. Foram usados testes t pareados para investigar alterações nas percepções dos participantes sobre os comportamentos em saúde e sentimentos antes e durante a pandemia da COVID-19. O teste ANOVA de mão única foi usado para testar as diferenças significativas entre necessidades de informação e níveis de letramento em saúde. Um p <0,05 foi considerado estatisticamente significativo para todos os testes.

## Resultados

### Características dos participantes

No total, 159 (25,5%) pacientes assinaram o formulário de consentimento informado e preencheram a pesquisa online. Os motivos para não participar incluíam os seguintes: 288 (46,2%) pacientes não atenderam a primeira ligação telefônica, 82 (13,2%) pacientes não foram encontrados devido a mudança no número de telefone, 64 (10,3%) pacientes não quiseram participar, 19 (3,0%) pacientes não se qualificaram, e 7 (1,8%) pacientes morreram. A Tabela 1 apresenta as características socioeconômicas e clínicas dos participantes.

**Tabela 1:** – Situação socioeconômica, características clínicas e letramento em saúde dos participantes (n=159)

Característica		Geral (n=159)	p*
**Sociodemográfica**			
Idade, média ± DP	-	62,7±10,1	-
Idade, n (%)	Abaixo de 65 anos	91 (57,2)	0,07
	65 anos ou mais	68 (42,8)	
Sexo, n (%)	Masculino	96 (60,4)	<0,001
	Feminino	62 (39,0)	
	Não informado	1 (0,6)	
Estado civil†, n (%)	Casado	106 (66,7)	<0,001
	Viúvo	21 (13,20)	
	Divorciado	18 (11,3)	
	Solteiro	13 (8,2)	
	Não informado	1 (0,6)	
Número de pessoas vivendo no mesmo domicílio, média ± DP	-	2,5±1,2	-
Pessoas vivendo sozinhas, n (%)	-	26 (16,4)	-
Nível de escolaridade, n (%)	Ensino fundamental ou menos	57 (35,8)	0,07
	Ensino médio	65 (40,9)	
	Ensino superior	37 (23,3)	
Renda familiar†, n (%)	Até 4 salários-mínimos por mês	106 (66,7)	<0,001
	Entre 5 e 10 salários-mínimos por mês	31 (19,5)	
	Acima de 10 salários-mínimos por mês	22 (13,8)	
Mudança na renda familiar devido à pandemia da COVID-19	Sem mudanças	87 (54,7)	<0,001
	Renda mais baixa	62 (39,0)	
	Renda mais alta	6 (3,8)	
	Não informado	4 (2,5)	
**Clínica**			
Indicação de RC, n (% sim)	Doença arterial coronariana	100 (62,9)	0,04
	Insuficiência cardíaca	90 (56,6)	0,10
	Infarto do miocárdio	87 (54,7)	0,23
	Intervenção coronária percutânea	84 (52,8)	0,47
	Angioplastia coronária transluminal percutânea	68 (42,8)	0,07
	Enxerto de bypass na artéria coronária	46 (28,9)	<0,001
	Doença arterial periférica	11 (6,9)	<0,001
Fatores de risco e comorbidades, n (% sim)	Hipertensão	107 (67,3)	<0,001
	Ex-fumante	85 (53,5)	0,05
	Dislipidemia	70 (44,0)	0,13
	Diabetes tipo II	37 (23,3)	<0,001
	Obesidade	31 (19,5)	<0,001
	Acidente vascular	15 (9,4)	<0,001
	Diabetes tipo I	9 (5,7)	<0,001
	Câncer	6 (3,8)	<0,001
	Marca-passo	5 (3,1)	<0,001
Tomando os medicamentos prescritos, n (% sim)	-	159 (100,0)	-
Duração da participação na RC antes de o programa ser fechado devido à COVID-19, n (% sim)	Menos de 1 ano	35 (22,0)	<0,001
	Mais de 1 ano	121 (75,1)	
	Não informado	3 (1,9)	
**Níveis de letramento**			
Letramento em saúde, média ± DP		13,2±2,5	-
Classificação de letramento em saúde‡, n (% sim)	Letramento em saúde limitado	87 (54,7)	<0,001
	Letramento em saúde marginal	56 (35,2)	
	Letramento em saúde adequado	15 (9,4)	
	Não informado	1 (0,6)	

RC: reabilitação cardíaca; DP: desvio padrão. *Análises qui-quadrado para variáveis categóricas. †A renda familiar no Brasil é caracterizada por salários-mínimos por mês. Um salário-mínimo corresponde a 1.100,00 reais ou 193,60 dólares americanos (abril/2021). ‡ Classificação do letramento em saúde: pontuações de 4 a 12 pontos indicam letramento em saúde limitado, de 13 a 16, letramento em saúde marginal, e de 17 a 20, letramento em saúde adequado.

Conforme demonstrado, nossa amostra era composta majoritariamente por indivíduos do sexo masculino, casados, com uma renda familiar mensal de até 4 salários-mínimos (sendo que nenhuma alteração de renda devido a COVID-19 foi relatada), com um diagnóstico de doença arterial coronariana e hipertensão. Todos os participantes estavam tomando medicamentos prescritos relacionados a sua doença cardíaca. A maioria dos participantes (75,0%) frequentaram a RC por mais de um ano antes de os programas serem fechados por causa da pandemia. Em relação ao letramento em saúde ( Tabela 1 ), os participantes tiveram uma pontuação média de 13,2±2,5, com a maioria da amostra (89,9%) sendo classificada como tendo letramento em saúde limitado ou marginal. Os resultados demonstraram uma correlação positiva significativa entre nível de escolaridade e letramento em saúde (r=0,45; p=<0,001).

Em relação ao uso de tecnologia, 153 (96,2%) participantes relataram que tinham acesso à internet em casa. Para aqueles que não tinham acesso à internet em casa, os motivos para isso são baixo conhecimento da tecnologia, preço e a não percepção da necessidade de tê-la (n=2; 1,3% cada). A maioria dos usuários de tecnologia (n=138; 86,8%) relataram que usam tecnologia móvel, sendo que os telefones celulares são a tecnologia mais comum usada em casa (n=137; 86,2%). Por último, 99 (62,3%) participantes indicaram que usam a internet para pesquisar informações relacionadas a sua condição de saúde. Não foram encontradas correlações significativas entre ter acesso à internet em casa e características socioeconômicas.

**Figura 1:**
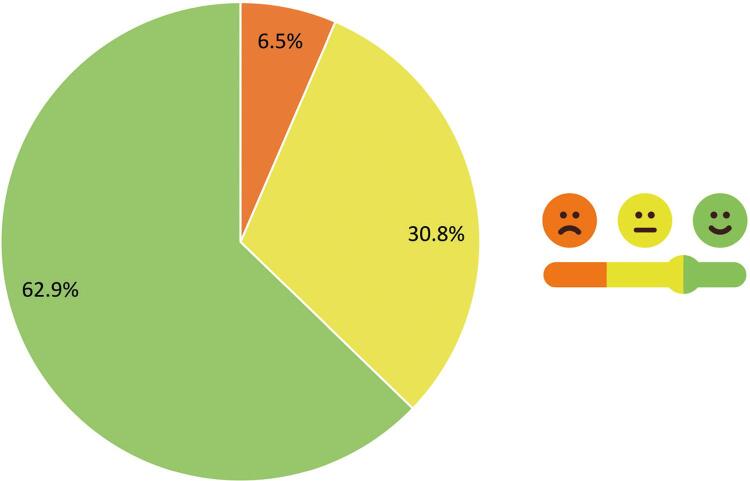
– Como os participantes percebem sua saúde.

A Figura 1 ilustra como os participantes percebem sua saúde em geral. Como demonstrado, a maioria dos participantes (n=100; 62,9%) achava que sua saúde era boa.

### Percepções sobre a pandemia da covid-19

Ao serem perguntados onde pesquisavam informações sobre a COVID-19, 135 (84,9%) participantes identificaram a televisão como principal fonte para conhecimento sobre a pandemia. Outras fontes incluíam o seguinte: familiares e amigos (n=87; 54,7%), jornais (n=59; 37,1%), redes sociais (n=59; 37,1%), e seus médicos (n=35; 22,0%). Além disso, medidas de segurança adotadas pelos participantes contra a COVID-19 incluíam o uso de máscara facial (n=155; 97,5%), distanciamento social (n=150; 94,3%), lavagem frequente das mãos (n=144; 90,6%), e uso de álcool em gel (n=60; 37,7%).

Ao serem perguntados sobre sua percepção em relação ao impacto da COVID-19 em seu problema cardíaco, 42 (26,4%) participantes relataram que achavam que a pandemia havia agravado seus sintomas. Os sintomas descritos eram os seguintes: dor torácica (n=13; 8,2%), falta de ar (n=13; 8,2%), cansaço (n=11; 6,9%), palpitação cardíaca (n=5; 3,1%), e dor no corpo (n=5; 3,1%). Ansiedade e depressão foram relatadas por 6 (3,8%) participantes.

**Figura 2:**
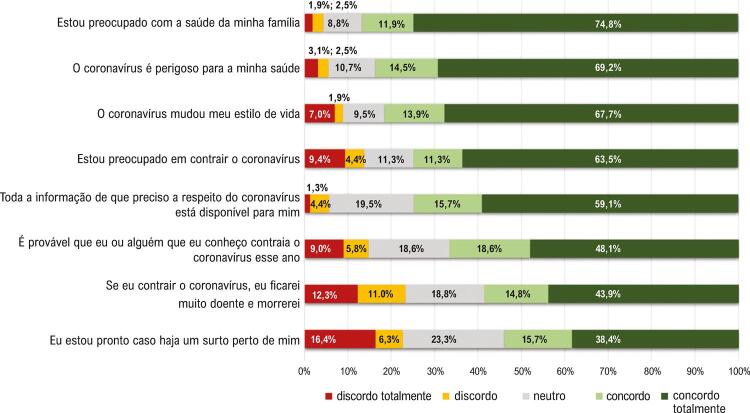
– Como os participantes da reabilitação cardíaca perceberam o impacto do COVID-19 em suas vidas.

A Figura 2 ilustra como os participantes de RC perceberam o impacto da COVID-19 em suas vidas, usando uma escala Likert que variava entre 1 = discordo totalmente e 5 = concordo totalmente. Os resultados mostram que os participantes estavam preocupados com a saúde de sua família (n=119; 74,8%), acham que o coronavírus é perigoso para sua saúde (n=110; 69,2%), mudaram o estilo de vida (n=107; 67,7%), e estão preocupados em contrair o coronavírus (n=101; 63,5%). Além disso, 94 (59,1%) participantes relataram que têm todas as informações de que precisam em relação ao coronavírus. Ademais, 75 (48,1%) participantes identificaram que é provável que eles (ou alguém que eles conhecem) vão contrair o coronavírus este ano, 68 (43,9%) acreditaram que, se contraíssem a doença, morreriam, e 61 (38,4%) participantes estão prontos para um surto. Os resultados também demonstraram uma correlação positiva entre letramento em saúde e as percepções relacionadas à morte devido à doença (r=0,29; p=0,01) e ter todas as informações relacionadas ao coronavírus (r=0,27; p=0,01), sendo que os participantes com letramento em saúde adequado perceberam a gravidade da doença e tinham acesso à informação.

### Percepções sobre os comportamentos em saúde e sentimentos antes e durante a pandemia da covid-19

A Tabela 2 apresenta uma comparação das percepções dos participantes sobre os comportamentos em saúde e sentimentos antes e durante a pandemia da COVID-19. No geral, os participantes perceberam que a qualidade de seus comportamentos em saúde diminuiu significativamente durante a pandemia, incluindo levar uma vida ativa (p<0,001), ter uma dieta saudável (p=0,04), dormir bem (p=0,04) e controlar níveis de ansiedade (p=0,01). Além disso, percebeu-se que a qualidade do nível de energia e entusiasmo para fazer mudanças em prol de um estilo de vida saudável diminuiu significativamente antes e durante a pandemia da COVID-19 (p<0,001), bem como sua percepção da saúde em geral (p=0,02).

**Tabela 2:** – Percepções dos participantes sobre comportamentos em saúde e sentimentos antes e durante a pandemia da COVID-19 (n=159)

Comportamentos em saúde e sentimentos	Como você classificaria este comportamento ou sentimento...	...antes da COVID-19?	... durante a COVID-19?	p*
Manter-se ativo, média ± DP		4,20±0,77	2,84±1,20	<0,001
	Ruim, n (%)	2 (1,3)	25 (15,7)	
	Razoável, n (%)	3 (1,9)	43 (27,0)	
	Neutro, n (%)	13 (8,2)	35 (22,0)	
	Bom, n (%)	85 (53,5)	45 (28,3)	
	Excelente, n (%)	56 (35,2)	11 (6,9)	
Ter alimentação saudável, média ± DP		4,17±0,61	4,01±0,94	0,04
	Ruim, n (%)	0 (0,0)	4 (2,5)	
	Razoável, n (%)	3 (1,9)	12 (7,5)	
	Neutro, n (%)	9 (5,7)	9 (5,7)	
	Bom, n (%)	105 (66,0)	87 (54,7)	
	Excelente, n (%)	42 (26,4)	47 (29,6)	
Dormir bem, média ± DP		3,66±0,95	3,35±1,19	0,04
	Ruim, n (%)	4 (2,5)	16 (10,1)	
	Razoável, n (%)	21 (13,2)	26 (16,4)	
	Neutro, n (%)	21 (13,2)	22 (13,8)	
	Bom, n (%)	91 (57,2)	76 (47,8)	
	Excelente, n (%)	22 (13,8)	19 (11,9)	
Controle de níveis de ansiedade, média ± DP		3,76±0,98	3,00±1,19	0,01
	Ruim, n (%)	2 (1,3)	18 (11,3)	
	Razoável, n (%)	22 (13,8)	40 (25,2)	
	Neutro, n (%)	22 (13,8)	42 (26,4)	
	Bom, n (%)	79 (49,7)	42 (26,4)	
	Excelente, n (%)	34 (21,4)	17 (10,7)	
Nível de energia e entusiasmo para fazer mudanças em prol de estilo de vida saudável, média ± DP		4,21±0,71	3,26±1,08	<0,001
	Ruim, n (%)	1 (0,6)	9 (5,7)	
	Razoável, n (%)	2 (1,3)	30 (18,9)	
	Neutro, n (%)	15 (9,4)	51 (32,1)	
	Bom, n (%)	85 (53,5)	49 (30,8)	
	Excelente, n (%)	56 (35,2)	20 (12,6)	
Percepção em relação à saúde geral, média ± DP		3,94±0,71	3,45±1,08	0,02
	Ruim, n (%)	0 (0,0)	8 (5,0)	
	Razoável, n (%)	6 (3,8)	25 (15,7)	
	Neutro, n (%)	27 (17,0)	33 (20,8)	
	Bom, n (%)	96 (60,4)	68 (42,8)	
	Excelente, n (%)	29 (18,2)	21 (13,2)	

DP: desvio padrão. *Testes t pareados usados como dados têm distribuição normal (p<0,05). Pontuações em escala Likert variando entre 1 = ruim e 5 = excelente.

Especificamente sobre atividade física, os participantes relataram as seguintes dificuldades de se manterem ativos durante a pandemia: falta de equipamento de exercício e um local físico para se exercitar (n=72; 45,3%), dificuldade de respirar usando máscara facial durante o exercício (n=63; 39,6), falta de motivação para se exercitar durante a pandemia (n=60; 37,7%), não ter o espaço físico adequado para se exercitar em casa (n=43; 27,0%), o uso de máscara facial que dificulta o exercício (n=63; 39,6), e a falta de orientação profissional para se exercitar com segurança (n=23; 14,5%).

### Necessidades de informação durante a pandemia da covid-19

**Figura 3:**
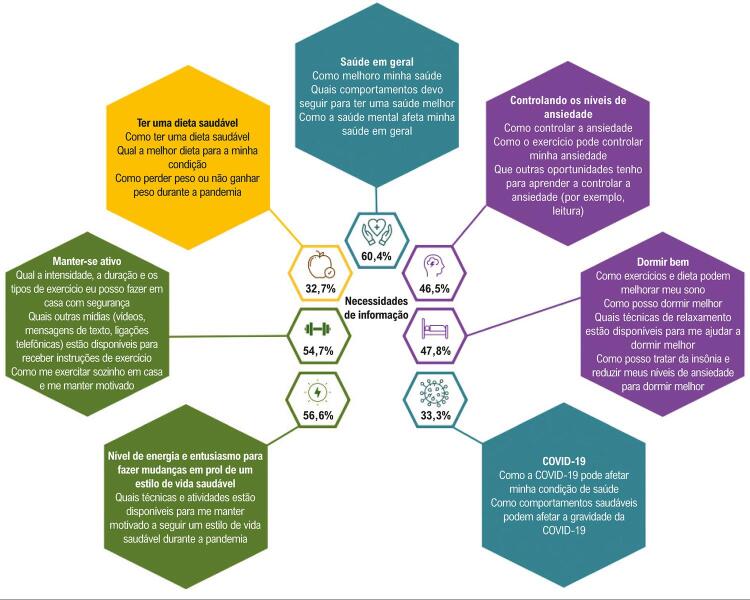
– Principais necessidades de informação percebidas pelos participantes durante a COVID-19.

A Figura 3 ilustra as principais necessidades de informação percebidas pelos participantes. As necessidades mais frequentes durante a pandemia estavam relacionadas à saúde em geral, ao nível de energia e o entusiasmo para fazer escolhas de estilo de vida saudáveis, e manter-se ativo. Ao serem perguntados sobre como prefeririam receber essas informações, 77 (48,4%) responderam que preferiam receber pelo WhatsApp, 26 (16,4%), por e-mail, e 7 (4,4%), pessoalmente; 49 (30,8%) participantes não responderam a essa pergunta.

Quando se solicitou que identificassem necessidades de informações específicas a tópicos educativos que podem ajudá-los a aderir a comportamentos saudáveis, a pontuação média foi 4,53±0,36, com os participantes fazendo pontuações acima de 4 (ou seja, importante) em todos os 12 tópicos educativos. O tópico com necessidade mais alta era “Tomar remédios”, e o mais baixo era “Iniciar um programa de treinamento de resistência” ( Tabela 3 ). Além disso, as necessidades de informações dos participantes eram significativamente diferentes dependendo dos níveis gerais de letramento em saúde (p=0,01) e em relação aos seguintes tópicos educativos: “Iniciar um programa de treinamento de resistência” (p=0,03), “Desenvolver uma relação saudável com a comida” (p=0,007), e “Controlar depressão, stress e burnout” (p=0,03).

**Tabela 3:** – Necessidades de informação específicas a tópicos de educação que poderiam ajudar pacientes a aderir a comportamentos saudáveis (n=159)

Tópico educacional	Pontuação média geral (média ± DP)	Pontuação média por nível de letramento em saúde (média ± DP)			p*

		Limitado (n=87)	Marginal (n=56)	Adequado (n=15)	
Criar um plano de mudança Descrição: aprenda a se motivar a ter uma vida saudável e como criar um plano de mudança que o ajudará a alcançar esse objetivo.	4,49±0,56	4,48±0,53	4,59±0,53	4,25±0,45	0,10
Iniciar um programa de exercícios aeróbicos Descrição: aprenda o que é exercício aeróbico, como planejar para se exercitar, os benefícios do exercício aeróbico e como se exercitar com segurança.	4,64±0,50	4,60±0,49	4,71±0,50	4,64±0,50	0,43
Iniciar um programa de treinamento de resistência Descrição: aprenda o que é treinamento de resistência e seus benefícios, e como fazer um treinamento de resistência com segurança.	4,12±0,88	4,12±0,91	4,27±0,80	3,57±0,94	0,03
Ficar menos sentado e movimentar-se mais Descrição: aprenda como ficar muito tempo sentado afeta sua saúde e quais são as formas para se sentar menos durante o dia.	4,44±0,61	4,39±0,65	4,50±0,57	4,43±0,51	0,59
Escolher alimentos saudáveis Descrição: aprenda que tipos de alimentos podem melhorar sua saúde cardíaca e como usar a tabela nutricional para escolher alimentos saudáveis.	4,65±0,55	4,61±0,54	4,76±0,54	4,60±0,51	0,22
Desenvolver uma relação saudável com a comida Descrição: aprenda a importância de se prestar atenção a sabor, textura e seu ambiente quando estiver comendo, e quais são as maneiras de se comer com mais prazer e saber quando está satisfeito.	4,57±0,55	4,57±0,50	4,69±0,47	4,20±0,51	0,007
Comer a dieta mediterrânea Descrição: Aprenda que alimentos incluir em um padrão de alimentação saudável para o coração e como incluir mais alimentos integrais em sua dieta.	4,56±0,59	4,54±0,57	4,60±0,63	4,64±0,50	0,73
Tomar remédios Descrição: saiba quais são as classes comuns de medicamentos para o coração, como eles o ajudam e quem pode ajudá-lo a controlar efeitos colaterais e responder às suas perguntas.	4,77±0,44	4,74±0,47	4,85±0,36	4,67±0,49	0,18
Controlar depressão, stress e burnout Descrição: aprenda o que são depressão, stress e burnout, e quais técnicas você pode tentar para ajudá-lo a se sentir responsável por sua saúde.	4,52±0,59	4,54±0,59	4,57±0,54	4,13±0,74	0,03
Dormir melhor Descrição: aprenda o que pode estar impedindo que você durma bem, e quais são os sinais de apneia do sono.	4,57±0,67	4,53±0,78	4,65±0,52	4,40±0,51	0,35
Fortalecer relações sociais Descrição: aprenda como as relações sociais podem melhorar sua saúde, como doenças cardíacas podem afetar sexo e intimidade, e que técnicas estão disponíveis para criar relações saudáveis.	4,30±0,76	4,31±0,74	4,36±0,78	4,14±0,77	0,62
Escolher ser saudável todos os dias Descrição: aprenda como manter seus hábitos saudáveis e o que fazer se você os interromper.	4,62±0,53	4,61±0,54	4,70±0,46	4,47±0,52	0,25
Total	4,53±0,36	4,51±0,37	4,63±0,31	4,29±0,36	0,01

DP: desvio padrão. *ANOVA de mão única (p<0,05). Pontuações em escala Likert variando entre 1 = ruim e 5 = excelente.

## Discussão

A pandemia da COVID-19 mudou significativamente os comportamentos em todo o mundo. Até onde sabemos, este é o primeiro estudo que examina as percepções dos participantes de RC em relação a seus comportamentos em saúde e necessidades de informação durante a pandemia da COVID-19 no Brasil, que foi feito em um dos países mais afetados por essa doença infecciosa no mundo. Os resultados confirmam que o impacto da COVID-19 vai além de aqueles que estão sofrendo da doença, e afeta não só a prestação do atendimento a doenças crônicas, como também os comportamentos e a saúde mental dos pacientes. Os pacientes se preocupam principalmente com a saúde de sua família e própria, além de como o coronavírus é perigoso para sua saúde e como mudou seu estilo de vida. No geral, os participantes perceberam que a qualidade de seus comportamentos em saúde diminuiu significativamente durante a pandemia. A pandemia também mudou as necessidades de informações dos participantes de RC; embora eles continuem interessados em aprender sobre como se manter ativo, dormir bem, e ter uma dieta saudável, novas necessidades de informação surgiram se comparadas a estudos anteriores com essa população.^28 , 29^ Este estudo identificou que os participantes de RC também precisam aprender sobre controle de níveis de ansiedade, o que pode ser feito para manter a motivação para levar uma vida saudável durante a pandemia, e como a COVID-19 pode afetar sua condição de saúde.

O letramento em saúde – as habilidades e competências das pessoas e das organizações em atender às demandas complexas de saúde na sociedade moderna^30^ – desempenha um papel central nesse cenário. O letramento em saúde limitado foi associado independentemente a uma menor utilização de serviços preventivos, maior uso de atendimento de emergência, internações hospitalares recorrentes, baixa qualidade de vida, alta ansiedade, apoio social mais baixo, condição de saúde geral pior e índices de mortalidade mais altos.^31 - 33^ Este estudo identificou que a maioria dos participantes tinha letramento em saúde limitado ou marginal, o que influenciou sua capacidade de lidar com as restrições da COVID-19. Participantes com letramento em saúde adequado perceberam significativamente a gravidade da doença e tinham significativamente mais acesso a informações do que os pacientes com letramento em saúde limitado. Além disso, aqueles com nível mais baixos de letramento em saúde tinham necessidades de informação mais altas do que os participantes com níveis adequados, que deveriam ser usados para informar a prática clínica. Há várias intervenções para mitigar o impacto do letramento em saúde inadequado;^34 , 35^ entretanto, as habilidades dos pacientes geralmente são superestimadas,^36^ e problemas, que raramente são identificados, poderiam ser aumentados em um cenário virtual.^37^ São necessárias maneiras eficientes de se incorporar a tecnologia da saúde a intervenções para participantes de RC com letramento em saúde limitado.

Os participantes deste estudo relataram que seu controle sobre níveis de ansiedade diminuiu significativamente durante a pandemia, e, além disso, eles perceberam que a pandemia piorou seus sintomas de ansiedade e depressão. Os efeitos adversos das restrições da COVID-19 sobre o bem-estar mental de pacientes foram observados e outros estudos.^38 - 40^ Como a ansiedade e a depressão são fatores conhecidamente associados a resultados de DCV piores,^41 , 42^ é essencial que os participantes de RC tenham o apoio relacionado à saúde psicológica durante esse tempo sem precedentes. Um dos canais de comunicação pode ser a educação, que pode tratar não apenas do impacto dos fatores psicossociais sobre a saúde, mas das implicações dessa pandemia na saúde mental na era pós-COVID.

A mídia tem um papel essencial em oferecer rotas de disseminação rápidas e eficientes para informações importantes durante a pandemia.^43 - 45^ Essa informação também foi confirmada em nosso estudo, uma vez que a maioria dos participantes de RC identificaram a televisão, os jornais e as redes sociais como as principais fontes de conhecimento relacionado à COVID-19. Embora as plataformas de mídia possam disseminar informação e educar as pessoas para que tomem medidas de saúde pública, elas também podem levar à desinformação, falta de orientação, e vazamento de informações.^44 , 46^ A necessidade das habilidades de julgar corretamente a precisão das informações de saúde postadas em canais de mídia coloca indivíduos com letramento em saúde limitado em risco de desinformação.^46^ Embora poucos participantes deste estudo tenham relatado que procuram seus médicos para obter informações relacionadas à COVID-19, as equipes de assistência de saúde devem incluir esses tópicos em suas sessões e, se possível, criar canais de redes sociais para se conectar a seus pacientes e compartilhar recomendações para os tempos de COVID-19.

A tecnologia é considerada uma forma segura para garantir que pacientes cardíacos recebam o cuidado de que precisam durante a pandemia.^13 , 15 , 26^ As percepções dos pacientes e as maiores necessidades de informação relatadas nesse estudo confirmam a urgência da assistência a esses pacientes. Estudos identificaram que a maioria dos componentes de RC poderiam ser oferecidos com segurança por meios remotos, incluindo a educação dos pacientes.^13 , 24 - 26 , 47 - 50^ Dessa forma, um meio de educação de pacientes virtual baseada em evidências e abrangente está disponível em 8 idiomas (incluindo o português brasileiro) para os programas usarem livremente. É necessário avaliar novos formatos de RC em implementação e resultados.

Este estudo articulou como a pandemia da COVID afetou as percepções de participantes de RC em relação a seus comportamentos em saúde e suas necessidades de informação, e a influência dos níveis de letramento em saúde nesse cenário. Indivíduos com letramento em saúde limitado enfrentam desafios para acessar e navegar pela assistência em saúde, e tais obstáculos podem ser exacerbados pelas restrições da pandemia. Entretanto, os resultados vão além do nível individual e são direcionados a prestadores de assistência de saúde e programas de RC. Os prestadores de assistência de saúde devem começar a adotar estratégias que têm potencial de mitigar o impacto do letramento em saúde no cuidado de seus pacientes. Os programas devem trabalhar para se tornar instituições letradas em saúde e desenvolver uma abordagem de boas práticas para o letramento em saúde.

É necessário ter cuidado ao se interpretar esses resultados. Primeiramente, essa era uma amostra de conveniência; portanto os resultados podem ter viés. A amostra era pequena, o que limita a possibilidade de generalização. Os resultados podem não se aplicar a outros grupos de pacientes cardíacos. Segundo, a confiabilidade e a validade do questionário não são conhecidas. Terceiro, este foi um estudo transversal, e, portanto, os dados foram capturados em um único momento no tempo em tópicos específicos. Como o surto de COVID-19 muda constantemente, com várias ondas e restrições, espera-se que as percepções e comportamentos autorrelatados possam mudar. Por último, o desenho do estudo pode limitar a descrição das percepções. Estudos qualitativos subsequentes vão aumentar nosso entendimento desse tópico. Sugere-se também que futuros estudos deveriam testar a validade deste estudo em outros grupos de pacientes e descrever a metodologia aplicada em detalhes.

## Conclusão

Concluindo, nossos resultados destacaram o impacto da pandemia nas percepções dos participantes de RC em relação a seus comportamentos em saúde e necessidades de informação, que podem ser influenciados pelos níveis de letramento em saúde. Achados deste estudo devem ser usados para informar programas de RC e estimular prestadores de atendimento de saúde a personalizar o cuidado preventivo, que pode acabar ajudando pacientes a passar por esse período difícil, ajudando-os a se manter saudáveis e evitar eventos recorrentes.

## * Material suplementar

Para informação adicional, por favor,clique aqui.
