# Supplementary material for: Percepções dos Participantes de Reabilitação Cardíaca sobre seus Comportamentos em Saúde e Necessidades de Informação durante a Pandemia COVID-19 no Brasil
Source: Arq Bras Cardiol. 2022 Mar 21;118(5):949–60. [Article in Portuguese] doi: 10.36660/abc.20210447 (PMC9368887; doi:10.36660/abc.20210447)
Supplement: Translation

# Translation: Perceptions of Cardiac Rehabilitation Participants Regarding their Health Behaviors and Information Needs during the COVID-19 Pandemic in Brazil

**Authors:** Gabriela L.M Ghisi, Rafaella Z. Santos, Andrea S. Korbes, Cícero Augusto de Souza, Marlus Karsten, Paul Oh, Magnus Benetti

**Affiliations:** 1 Canada Cardiovascular Prevention and Rehabilitation Program, Toronto Rehabilitation Institute, University Health Network, Toronto – Canada; 2 Brazil Núcleo de Cardioncologia e Medicina do Exercício, Centro de Ciências da Saúde e do Esporte, Universidade do Estado de Santa Catarina, Florianópolis, SC – Brazil; 3 Brazil Instituto de Cardiologia de Santa Catarina, São José, SC – Brazil

**Keywords:** Cardiac Rehabilitation, COVID-19, Health Literacy, Needs Assessment, Surveys and Questionnaires

## Abstract

**Background:**

COVID-19 has impacted how people receive health care for many conditions, including cardiovascular diseases.

**Objectives:**

To examine perceptions of cardiac rehabilitation (CR) participants regarding their health behaviors and information needs during the COVID-19 pandemic in Brazil.

**Methods:**

In this cross-sectional study, a 27-item questionnaire, developed by the investigators, was administered online to participants from two CR programs. Questions included health literacy (HL; using the Brief Health Literacy Screening Tool), technology use, perceptions before and during the COVID-19 pandemic, and information needs. Pearson correlation coefficients, paired t-tests, and ANOVA were used as appropriate. P < 0.05 was considered statistically significant for all tests.

**Results:**

Overall, 159 (25.5%) CR participants answered the questionnaire. Of these, 89.9% had limited or marginal HL and 96.2% reported having internet access at home. Patients are mainly concerned about their family’s health and their own, as well as how the coronavirus is dangerous to their health and how it has changed their lifestyle. Participants perceived that the quality of their health behaviors significantly decreased during the pandemic. The pandemic also changed information needs of CR participants as new needs emerged, such as the control of anxiety levels, staying motivated to live healthily during a pandemic, and how COVID-19 can impact their health condition. Participants with adequate HL significantly perceived the severity of the disease and having access to information significantly more than those with limited HL.

**Conclusions:**

Our results highlighted the impact of the pandemic on CR participants’ perceptions regarding their health behaviors and information needs, which can be influenced by HL levels.

## Introduction

SARS-CoV-2 is a novel coronavirus identified as the cause of the coronavirus disease 2019 (COVID-19), which began in Wuhan, China in late 2019 and spread worldwide.^1^ More than one year after being declared a pandemic, the number of confirmed COVID-19 cases worldwide reached 147,000,000, with Brazil ranking third among the countries with the highest number of confirmed cases and second in number of deaths.^2^ Due to its *highly contagious* pathogenic, people worldwide are trying to prevent the spread of infection by practicing social distancing,^3^ which has impacted how they work, connect with others, and receive health care from many conditions, including cardiovascular diseases (CVDs).^4^

Cardiovascular diseases are among the leading burdens of disease and the leading cause of death worldwide, with more than 80% of these deaths occurring in low- and middle-income countries,^5^ including Brazil.^6^ Cardiac rehabilitation (CR) is an established model of secondary prevention that has not only proven clinical and cost-effectiveness, but can significantly reduce hospitalizations and mortality rates.^7 - 9^ In general, CR is delivered in clinical settings with patients visiting hospitals or rehabilitation centers for weekly in-person exercise and education sessions.^10 , 11^ Thus, the necessary measures to curb the widespread transmission of COVID-19 have affected the delivery of CR, with an estimation of approximately 4,400 programs worldwide closed due to COVID-19 and face-to-face services suspended.^12^

In Brazil, COVID-19 has affected an already suboptimal CR system,^11^ and programs have developed remote and innovative ways to deliver core components in such a delicate time,^12 , 13^ following local guidelines and recommendations.^14^ The rapid speed in which these changes occurred, the economic threats experienced by healthcare providers and their programs, and the inability to navigate the virtual world by many patients have affected CR participants in ways not yet explored. Although there are many publications on the COVID-19 impact on this population,^15 , 16^ to the best of our knowledge there are no studies on how CR participants perceive their health behaviors and what information they need to know in order to continue or adopt behaviors that will make them have a better health. This is particularly important as social distancing, quarantine, and stay-at-home orders impact our lifestyle and, in cardiac patients who are already sedentary and with risk factors due to poor behaviors,^17 , 18^ these measures can increase the risk of acute events. Furthermore, the indirect effects of the COVID-19 pandemic on general mental health are of increasing concern,^19 - 21^ mainly in individuals with cardiovascular conditions, since they are more likely to experience mental health problems (such as depression),^22^ which is associated with a two-fold higher risk of cardiovascular mortality.^23^

Therefore, there is an urgent need to monitor cardiac patients virtually and personalize prevention care, helping these individuals in their recovery and in preventing recurrent events.^24 - 26^ In order to design an optimum CR program during the COVID-19 pandemic and beyond, it is important to understand patients’ perceptions and needs. Thus, the objective of this study was to examine the perceptions of CR participants regarding their health behaviors and information needs during the COVID-19 pandemic in Brazil.

## Methods

### Design

This was a cross-sectional study in design. Ethics approval was obtained from the Human Research Ethics Committee of the State University of Santa Catarina (UDESC; Florianopolis, Brazil: 4.341.132). Data was collected between December/2020 and April/2021.

### Setting and Participants

A convenience sample of CR participants were recruited from two public programs in the Greater Florianopolis Area (Cardiology Institute of Santa Catarina and Cardio-Oncology and Exercise Medicine Program). Before the pandemic, patients used to go to these centers 3 times a week for 1-hour exercise sessions supervised by a multidisciplinary team. Some of these participants were also attending educational sessions as part of a research project. Due to COVID-19, both programs have been closed since March 2020, and activities were not resumed during this research. The exclusion criteria were the following: being illiterate, and any visual or cognitive condition that would preclude the participant from completing the survey.

### Procedures

There were 623 CR participants when both programs were closed due to COVID-19. All of them were contacted by phone and invited to participate in this research. Those interested were scheduled a second call to provide informed consent via video, which was recorded as indicated by the Research Ethics Board. Participants completed the survey online using *Google Docs* during a video chat with a research team member.

### Measures

A 27-item questionnaire was developed by the investigators to examine the objectives of this study (Appendix 1). The questionnaire was divided into 5 sections as follows: (1) sociodemographic characteristics, (2) health literacy and technology use, (3) perceptions about the COVID-19 pandemic, (4) perceptions about health behaviors and feelings before and during the COVID-19 pandemic, and (5) information needs during the COVID-19 pandemic.

The items had single-, multiple-choice, and open-ended response options. Perceptions about the pandemic were reported using a Likert-type scale ranging from 1=totally disagree to 5=totally agree. Perceptions about health behaviors and feelings before and during the COVID-19 pandemic were reported using a Likert-type scale ranging from 1=poor to 5=excellent. Information needs specific to educational topics that can help patients adhere to healthy behaviors were reported using a Likert-type scale ranging from 1=really not important to 5=very important; a mean score was computed and analyzed by literacy levels, with higher scores indicating higher information needs. Input from CR experts was solicited before conducting the survey.

Clinical data (CR referral indication and cardiac risk factors) was extracted from medical records and sociodemographic characteristics (level of education, family income, change in family income due to COVID-19, marital status, and number of people living in the same household) were self-reported by participants. Health literacy was assessed using the Brief Health Literacy Screening Tool,^27^ which was translated to Portuguese by the research team. Each one of the 4 items was worth 1 to 5 points, depending on participants’ responses, which could range from 4 to 20. Total scores from 4 to 12 were classified as limited health literacy, 13 to 16 as marginal health literacy, and 17 to 20 as adequate health literacy.

### Data analysis

Statistical analysis was performed using SPSS Version 27.0 (IBM Inc 2020, NYC). Descriptive statistics were used to describe participants’ socioeconomic and clinical characteristics. Continuous variables were presented as mean and standard deviation and categorical variables by absolute numbers and percentages. Chi-square analysis for categorical variables and t-tests for continuous variables were used to compare proportions of respondents across different characteristics. All open-ended responses were coded. Pearson correlation coefficients were used to determine the association between health literacy and educational level, the use of technology and socioeconomic characteristics, and health literacy and perceptions about the COVID-19 pandemic.

The normality of data distribution was tested using the *Kolmogorov Smirnov* test. Paired t-tests were used to investigate changes between participants’ perceptions of health behaviors and feelings before and during the COVID-19 pandemic. ANOVA One-Way was used to test for significant differences between information needs and health literacy levels. P < 0.05 was considered statistically significant for all tests.

## Results

### Participants’ characteristics

Overall, 159 (25.5%) patients signed the consent form and completed the online survey. Reasons for non-participation included the following: 288 (46.2%) patients did not answer the first phone call, 82 (13.2%) patients were not reached due to a change in phone number, 64 (10.3%) patients did not want to participate, 19 (3.0%) patients were not eligible, and 7 (1.8%) patients died. Table 1 presents the socioeconomic and clinical characteristics of participants.

**Table 1:** – Socioeconomic status, clinical characteristics, and health literacy of participants (n=159)

Characteristic		Overall (n=159)	p*
Sociodemographic			
Age, mean±SD	-	62.7±10.1	-
Age, n (%)	Less than 65 years old	91 (57.2)	0.07
	65 years old or older	68 (42.8)	
Sex, n (%)	Male	96 (60.4)	<0.001
	Female	62 (39.0)	
	Missing	1 (0.6)	
Marital status†, n (%)	Married	106 (66.7)	<0.001
	Widower/widow	21 (13.20	
	Divorced	18 (11.3)	
	Single	13 (8.2)	
	Missing	1 (0.6)	
Number of people living in the same household, mean±SD	-	2.5±1.2	-
People living alone, n (%)	-	26 (16.4)	-
Level of education, n (%)	Elementary school or less	57 (35.8)	0.07
	High school	65 (40.9)	
	University degree	37 (23.3)	
Family income†, n (%)	Under or equal to 4 minimum wages per month	106 (66.7)	<0.001
	Between 5 and 10 minimum wages per month	31 (19.5)	
	Above 10 minimum wages per month	22 (13.8)	
Change in family income due to the COVID-19 pandemic	No change	87 (54.7)	<0.001
	Lower income	62 (39.0)	
	Higher income	6 (3.8)	
	Missing	4 (2.5)	
**Clinical**			
CR referral indication, n (% yes)	Coronary Artery Disease	100 (62.9)	0.04
	Heart Failure	90 (56.6)	0.10
	Myocardial Infarction	87 (54.7)	0.23
	Percutaneous Coronary Intervention	84 (52.8)	0.47
	Percutaneous Transluminal Coronary Angioplasty	68 (42.8)	0.07
	Coronary Artery Bypass Grafting	46 (28.9)	<0.001
	Peripheral Arterial Disease	11 (6.9)	<0.001
Risk factors and comorbidities, n (% yes)	Hypertension	107 (67.3)	<0.001
	Former smoker	85 (53.5)	0.05
	Dyslipidemia	70 (44.0)	0.13
	Diabetes Type II	37 (23.3)	<0.001
	Obesity	31 (19.5)	<0.001
	Stroke	15 (9.4)	<0.001
	Diabetes Type I	9 (5.7)	<0.001
	Cancer	6 (3.8)	<0.001
	Pacemaker	5 (3.1)	<0.001
Taking prescribed medications, n (% yes)	-	159 (100.0)	-
Duration of participation in CR before the program was closed due to COVID-19, n (% yes)	Less than 1 year	35 (22.0)	<0.001
	More than 1 year	121 (75.1)	
	Missing	3 (1.9)	
**Literacy levels**			
Health literacy, mean±SD		13.2±2.5	-
Health literacy classification‡, n (% yes)	Limited health literacy	87 (54.7)	<0.001
	Marginal health literacy	56 (35.2)	
	Adequate health literacy	15 (9.4)	
	Missing	1 (0.6)	

CR: cardiac rehabilitation; SD: standard deviation. *Chi-square analyses for categorical variables. †Family income in Brazil is characterized by minimum wages per month. One minimum wage is 1,100.00 BRL or 193.60 USD (April/2021). ‡ Health literacy classification: total scores from 4 to 12 indicate limited health literacy, 13 to 16 marginal health literacy, and 17 to 20 adequate health literacy.

As shown, our sample was consisted mainly of male individuals, married, with a monthly family income of 4-times the Brazilian minimum wage or lower per month (with no reported changes of income due to COVID-19), with a diagnosis of coronary artery disease and hypertension. All participants were taking prescribed medications related to their cardiac condition. Most participants (75.0%) attended CR for more than one year before programs were closed due to the pandemic. As regards health literacy ( Table 1 ), participants presented a mean score of 13.2±2.5, with the majority of the sample (89.9%) classified as having limited or marginal health literacy. Results also showed a significant positive correlation between educational level and health literacy (r=0.45; P=<0.001).

Regarding technology use, 153 (96.2%) participants reported that they have internet access at home. For those who do not have internet access at home, their reasons for not having are low technological literacy, price, and not perceiving the need to have it (n=2; 1.3% each). Most technology users (n=138; 86.8%) reported using mobile technology, with mobile phones being the most common single technology used at home (n=137; 86.2%). Finally, 99 (62.3%) participants indicated they use the internet to search for information regarding their health condition. No significant correlations between having internet access at home and socioeconomic characteristics were found.

Figure 1 illustrates how participants perceive their overall health. As shown, most participants (n=100; 62.9%) felt their health was good.

**Figure 1:** – How participants perceive their overall health.

### Perceptions about the covid-19 pandemic

When asked where they search for information about COVID-19, 135 (84.9%) participants identified the television as the main source for knowledge regarding the pandemic. Other sources include the following: family and friends (n=87; 54.7%), newspaper (n=59; 37.1%), social media (n=59; 37.1%), and their doctors (n=35; 22.0%). Furthermore, safety measures adopted by participants against COVID-19 included the use of facial masks (n=155; 97.5%), social distancing (n=150; 94.3%), frequent hand washing (n=144; 90.6%), and the use of hand sanitizer (n=60; 37.7%).

When asked about their perception regarding the impact of COVID-19 on their cardiac condition, 42 (26.4%) participants reported they felt the pandemic has aggravated their symptoms. Described symptoms were the following: chest pain (n=13; 8.2%), shortness of breath (n=13; 8.2%), tiredness (n=11; 6.9%), heart palpitations (n=5; 3.1%), and body pain (n=5; 3.1%). Anxiety and depression were reported by 6 (3.8%) participants.

Figure 2 illustrates how CR participants perceived the impact of COVID-19 on their lives using a Likert-type scale ranging from 1=totally disagree to 5=totally agree. Results revealed that participants were worried about their family’s health (n=119; 74.8%), think that the coronavirus is dangerous to their health (n=110; 69.2%) and changed their lifestyle (n=107; 67.7%), and are worried about catching the coronavirus (n=101; 63.5%). In addition, 94 (59.1%) participants reported they have all the information they need regarding the coronavirus. Furthermore, 75 (48.1%) participants identified that it is likely they (or someone they know) will catch the coronavirus this year, 68 (43.9%) believed that if they get the disease they will die, and 61 (38.4%) participants are ready for an outbreak. Results also showed a significant positive correlation between health literacy and perceptions related to dying from this disease (r=0.29; p=0.01) and having all the information they need regarding the coronavirus (r=0.27; p=0.01), with participants with adequate health literacy perceiving the severity of the disease and having access to information.

**Figure 2:** – How CR participants perceived the impact of COVID-19 on their lives.

### Perceptions about health behaviors and feelings before and during the covid-19 pandemic

Table 2 presents a comparison of participants’ perceptions about health behaviors and feelings before and during the COVID-19 pandemic. Overall, participants perceived that the quality of health behaviors significantly decreased during the pandemic, including being active (p<0.001), eating a healthy diet (p=0.04), sleeping well (p=0.04), and controlling anxiety levels (p=0.01). In addition, the quality of the energy level and enthusiasm to make healthy lifestyle changes was perceived to decrease significantly before and during the COVID-19 pandemic (p<0.001), as was their perception about overall health (p=0.02).

**Table 2:** – Participants’ perceptions of health behaviors and feelings before and during the COVID-19 pandemic (n=159)

Health behaviors and feelings	How would you classify this behavior or feeling…	…before COVID-19?	…during COVID-19?	p*
Being active, mean±SD		4.20±0.77	2.84±1.20	<0.001
	Poor, n (%)	2 (1.3)	25 (15.7)	
	Fair, n (%)	3 (1.9)	43 (27.0)	
	Neutral, n (%)	13 (8.2)	35 (22.0)	
	Good, n (%)	85 (53.5)	45 (28.3)	
	Excellent, n (%)	56 (35.2)	11 (6.9)	
Eating a healthy diet, mean±SD		4.17±0.61	4.01±0.94	0.04
	Poor, n (%)	0 (0.0)	4 (2.5)	
	Fair, n (%)	3 (1.9)	12 (7.5)	
	Neutral, n (%)	9 (5.7)	9 (5.7)	
	Good, n (%)	105 (66.0)	87 (54.7)	
	Excellent, n (%)	42 (26.4)	47 (29.6)	
Sleeping well, mean±SD		3.66±0.95	3.35±1.19	0.04
	Poor, n (%)	4 (2.5)	16 (10.1)	
	Fair, n (%)	21 (13.2)	26 (16.4)	
	Neutral, n (%)	21 (13.2)	22 (13.8)	
	Good, n (%)	91 (57.2)	76 (47.8)	
	Excellent, n (%)	22 (13.8)	19 (11.9)	
Controlling anxiety levels, mean±SD		3.76±0.98	3.00±1.19	0.01
	Poor, n (%)	2 (1.3)	18 (11.3)	
	Fair, n (%)	22 (13.8)	40 (25.2)	
	Neutral, n (%)	22 (13.8)	42 (26.4)	
	Good, n (%)	79 (49.7)	42 (26.4)	
	Excellent, n (%)	34 (21.4)	17 (10.7)	
Energy level and enthusiasm to make healthy lifestyle changes, mean±SD		4.21±0.71	3.26±1.08	<0.001
	Poor, n (%)	1 (0.6)	9 (5.7)	
	Fair, n (%)	2 (1.3)	30 (18.9)	
	Neutral, n (%)	15 (9.4)	51 (32.1)	
	Good, n (%)	85 (53.5)	49 (30.8)	
	Excellent, n (%)	56 (35.2)	20 (12.6)	
Perception about overall health, mean±SD		3.94±0.71	3.45±1.08	0.02
	Poor, n (%)	0 (0.0)	8 (5.0)	
	Fair, n (%)	6 (3.8)	25 (15.7)	
	Neutral, n (%)	27 (17.0)	33 (20.8)	
	Good, n (%)	96 (60.4)	68 (42.8)	
	Excellent, n (%)	29 (18.2)	21 (13.2)	

SD: standard deviation. *Paired t-tests used as data is normally distributed (p<0.05). Likert-type scores ranged from 1=poor to 5=excellent.

Specifically about physical activity, participants reported the following difficulties related to being active during the pandemic: lack of exercise equipment and a physical location to exercise (n=72; 45.3%), difficulty to breath while using the facial mask during training (n=63; 39.6), lack of motivation to exercise during a pandemic (n=60; 37.7%), not having the adequate physical space to exercise at home (n=43; 27.0%), use of facial mask which makes it difficult to exercise (n=63; 39.6), and lack of professional guidance to exercise safely (n=23; 14.5%).

### Information needs during the Covid-19 pandemic

Figure 3 illustrates the main information needs perceived by participants. The most frequent needs during the pandemic were related to overall health, energy level, and enthusiasm to make healthy lifestyle choices, as well as being active. When asked how they would prefer this information to be delivered to them, 77 (48.4%) responded by WhatsApp, 26 (16.4%) by email, and 7 (4.4%) in person; 49 (30.8%) participants did not answer this question.

**Figure 3:** – Main information needs perceived by participants during COVID-19.

When asked to identify their information needs specific to educational topics that can help them adhere to healthy behaviors, the mean score was 4.53±0.36, with participants rating scores higher than 4 (i.e. important) in all 12 educational topics. The topic with the highest need was “Take medicines” and the lowest was “Start a resistance training program” ( Table 3 ). In addition, information needs of participants were significantly different between health literacy levels overall (p=0.01) and in regards to the following educational topics: “Start a resistance training program” (p=0.03); “Develop a healthy relationship with food” (p=0.007); and “Manage depression, stress, and burnout” (p=0.03).

**Table 3:** – Information needs specific to educational topics that can help patients adhere to healthy behaviors (n=159)

Educational topic	Mean score overall (mean±SD)	Mean score by health literacy level (mean±SD)			p*

		Limited (n=87)	Marginal (n=56)	Adequate (n=15)	
Create a plan for change Description: learn how to motivated oneself to live a healthy life and how to create a plan for change that will help you to reach this goal.	4.49±0.56	4.48±0.53	4.59±0.53	4.25±0.45	0.10
Start an aerobic exercise program Description: learn what aerobic exercise is, how to plan for exercise, the benefits of aerobic exercise and how to exercise safely.	4.64±0.50	4.60±0.49	4.71±0.50	4.64±0.50	0.43
Start a resistance training program Description: learn what resistance training is and its benefits and how to do resistance training safely.	4.12±0.88	4.12±0.91	4.27±0.80	3.57±0.94	0.03
Sit less and move more Description: learn how sitting too much affects your health and what are the ways to sit less during the day.	4.44±0.61	4.39±0.65	4.50±0.57	4.43±0.51	0.59
Choose healthy foods Description: learn what types of foods can improve your heart health and how to use a nutrition facts table to choose healthy foods.	4.65±0.55	4.61±0.54	4.76±0.54	4.60±0.51	0.22
Develop a healthy relationship with food Description: learn the importance of paying attention to flavor, texture, and your surroundings when you eat and what are the ways to eat with more pleasure and know when you are full.	4.57±0.55	4.57±0.50	4.69±0.47	4.20±0.51	0.007
Eat the Mediterranean diet Description: Learn what foods to include in a heart healthy eating pattern and how to include more whole foods in your eating.	4.56±0.59	4.54±0.57	4.60±0.63	4.64±0.50	0.73
Take medicines Description: learn what are the common classes of heart medicines, how they help you and who can help you manage side effects and answer your questions.	4.77±0.44	4.74±0.47	4.85±0.36	4.67±0.49	0.18
Manage depression, stress, and burnout Description: learn what depression, stress, and burnout are, and what techniques you can try to help you feel in charge of your health.	4.52±0.59	4.54±0.59	4.57±0.54	4.13±0.74	0.03
Sleep well Description: learn what might be stopping you from sleeping well and what are the signs of sleep apnea.	4.57±0.67	4.53±0.78	4.65±0.52	4.40±0.51	0.35
Strengthen social relationships Description: learn how social relationships can improve your health, how heart disease can affect sex and intimacy, and what techniques are available to create healthy relationships.	4.30±0.76	4.31±0.74	4.36±0.78	4.14±0.77	0.62
Choose health everyday Description: learn how to maintain your healthy habits and what to do if you stop your healthy habit.	4.62±0.53	4.61±0.54	4.70±0.46	4.47±0.52	0.25
Total	4.53±0.36	4.51±0.37	4.63±0.31	4.29±0.36	0.01

SD: standard deviation. *ANOVA One-Way (p<0.05). Likert-type scores ranged from 1=poor to 5=excellent.

## Discussion

The COVID-19 pandemic has substantially changed behaviors around the globe. To the best of our knowledge, this is the first study examining perceptions of CR participants regarding their health behaviors and information needs during the COVID-19 pandemic, which was conducted in one of the countries most affected by this infectious disease in the world. Results confirm that the impact of COVID-19 goes well beyond those suffering from it, affecting not only the delivery of chronic disease care, but also patients’ behaviors and mental health. Patients are mainly concerned about their family’s health and their own, as well as how the coronavirus is dangerous to their health and how it has changed their lifestyle. Overall, participants perceived that the quality of their health behaviors significantly decreased during the pandemic. The pandemic also changed information needs of CR participants; although they continue to be interested to learn about being active, sleeping well, and eating a healthy diet, new information needs emerged when compared to previous studies with this population.^28 , 29^ This study identified that CR participants are now also in need of learning about controlling their anxiety levels, what they can do to motivated themselves to live healthily during a pandemic, and how COVID-19 can impact their health condition.

Health literacy – the skills and competences of people and organizations to meet the complex demands of health in modern society^30^ – plays a key role in this scenario. Limited health literacy has been independently associated with lesser use of preventive services, a greater use of emergency care, more hospital readmissions, a low quality of life, higher anxiety, lower social support, poorer overall health status, and higher mortality rates.^31 - 33^ This study has identified that the majority of participants had limited or marginal health literacy, which has influenced their ability to deal with COVID-19 restrictions. Participants with adequate health literacy perceived the severity of the disease and having access to information significantly more than those with limited health literacy. In addition, those with lower levels of health literacy had higher information needs than participants with adequate levels, which should be used to inform clinical practice. There are multiple interventions to mitigate the impact of inadequate health literacy;^34 , 35^ however, patients’ abilities are often overestimated,^36^ and problems, which are rarely identified, could be increased in the virtual setting.^37^ Effective ways to incorporate health technology in interventions for CR participants with limited health literacy are needed.

Participants of this study have reported that their control over anxiety levels has significantly decreased during the pandemic; in addition, they perceived that the pandemic has aggravated their symptoms of anxiety and depression. The adverse effects of COVID-19 restrictions on mental well-being in patients were noted by other studies.^38 - 40^ Because anxiety and depression are well-known factors associated with poorer outcomes of CVD,^41 , 42^ it is essential that CR participants receive support related to psychological health during this unprecedented time. One of the channels for communication can be education, which can address not only the impact of psychosocial factors on health, but the mental health implications of this pandemic into the post-COVID era.

The media plays a critical role in providing rapid and effective dissemination routes for key information during the pandemic.^43 - 45^ This information has also been confirmed in our study, as most CR participants identified television, newspaper, and social media as the main source for knowledge regarding COVID-19. Although media platforms can disseminate information and educate people to take public health measures, it can also lead to misinformation, a lack of guidance, and information leakage.^44 , 46^ The need for skills to correctly judge the accuracy of health information posted on media channels makes it individuals with limited health literacy at risk of misinformation.^46^ Although few participants of this study have reported they seek their doctors for information related to COVID-19, healthcare teams should include these topics to their sessions and, if possible, create social media channels to connect with their patients and share recommendations in times of COVID-19.

Technology is considered a safe way to ensure cardiac patients receive the care they need during the pandemic.^13 , 15 , 26^ Patients perceptions and higher information needs reported by this study confirm the urge to care for these patients. Studies have identified that most CR components could be safely delivered through remote means, including patient education.^13 , 24 - 26 , 47 - 50^ Thus, a comprehensive, evidence-based virtual patient education is available in 8 languages (including Brazilian-Portuguese) for programs to use freely. The assessment of new formats of CR on implementation and outcomes is needed.

This study has articulated how the COVID pandemic has impacted CR participants’ perceptions regarding their health behaviors and information needs, and the influence of health literacy levels in this scenario. Individuals with limited health literacy face challenges in accessing and navigating health care, and such obstacles may be exacerbated by pandemic restrictions. However, results go beyond the individual level and are also targeted to healthcare providers and CR programs. Healthcare providers should start adopting strategies that can potentially mitigate the impact of health literacy in the care of their patients. CR programs should work towards becoming health-literate institutions and develop a best practices approach to health literacy.

Caution is warranted when interpreting these results. First, this was a convenience sample; thus, results may be biased. This was a small sample size, which limits generalizability. Results may not be applicable to other groups of cardiac patients. Second, the reliability and validity of the questionnaire is unknown. Third, this was a cross-sectional study, so data was captured at a single moment in time on specific topics. Since the surge of COVID-19 has changed constantly with different waves and restrictions, it is expected that self-reported perceptions and behaviors could change. Third, the study design may limit the description of perceptions. Subsequent qualitative studies will increase our understanding of this topic. It is also suggested that future studies should test the validity of this study in other groups of patients and describe the methodology applied in detail.

## Conclusion

In conclusion, our results highlighted the impact of the pandemic on CR participants’ perceptions regarding their health behaviors and information needs, which can be influenced by health literacy levels. Findings from this study should be used to inform CR programs and encourage healthcare providers to personalize prevention care, which can ultimately help patients to navigate through such a difficult period, helping them to stay healthy and prevent recurrent events.

## * Supplemental Materials

For additional information, please click here.
